# Optical trapping and manipulating with a transmissive and polarization-insensitive metalens

**DOI:** 10.1515/nanoph-2023-0850

**Published:** 2024-04-15

**Authors:** Dongni Yang, Jianchao Zhang, Pengshuai Zhang, Haowen Liang, Jie Ma, Juntao Li, Xue-Hua Wang

**Affiliations:** State Key Laboratory of Optoelectronic Materials and Technologies, School of Physics, 26469 Sun Yat-Sen University, Guangzhou 510275, China; Quantum Science Center of Guangdong-Hong Kong-Macao Greater Bay Area, Shenzhen-Hong Kong International Science and Technology Park, No.3 Binglang Road, Futian District, Shenzhen, China; Hisense Laser Display Co., Ltd., Qingdao, China

**Keywords:** optical tweezers, metalens, polarization-insensitive

## Abstract

Trapping and manipulating micro-objects and achieving high-precision measurements of tiny forces and displacements are of paramount importance in both physical and biological research. While conventional optical tweezers rely on tightly focused beams generated by bulky microscope systems, the emergence of flat lenses, particularly metalenses, has revolutionized miniature optical tweezers applications. In contrast to traditional objectives, the metalenses can be seamlessly integrated into sample chambers, facilitating flat-optics-based light manipulation. In this study, we propose an experimentally realized transmissive and polarization-insensitive water-immersion metalens, constructed using adaptive nano-antennas. This metalens boasts an ultra-high numerical aperture of 1.28 and achieves a remarkable focusing efficiency of approximately 50 % at a wavelength of 532 nm. Employing this metalens, we successfully demonstrate stable optical trapping, achieving lateral trapping stiffness exceeding 500 pN/(μm W). This stiffness magnitude aligns with that of conventional objectives and surpasses the performance of previously reported flat lenses. Furthermore, our bead steering experiment showcases a lateral manipulation range exceeding 2 μm, including a region of around 0.5 μm exhibiting minimal changes in stiffness for smoothly optical manipulation. We believe that this metalens paves the way for flat-optics-based optical tweezers, simplifying and enhancing optical trapping and manipulation processes, attributing ease of use, reliability, high performance, and compatibility with prevalent optical tweezers applications, including single-molecule and single-cell experiments.

## 1 Introduction

Optical tweezers or traps (OTs) are optomechanical tools to trap and manipulate the micro-objects by a tightly focused laser beam. Since its inception in 1980s [1], OTs have been widely used in physical and biological researches because of their low damage, non-physical contact of objects, and high precision [2–5]. However, traditional OTs rely on bulky optical elements and long optical paths, making them difficult to be integrated into a compact platform and often suffer from severe drifting noise [6]. Several optical methods have been reported to effectively reduce the drifting noise [7], [8], but at the expense of more complicated configurations. In this case, lab-on-a-chip optical trapping devices [9]–[12] with portability and convenience are emerging. Since the objective is the key element in a conventional OT, it is essential to minimize its size before miniaturizing the whole system. To achieve this goal, the flat lenses have become one of the promising choices. The flat lenses, e.g. metalenses [13–17] and Fresnel zone plates [18]–[20], are thin film optical elements that control the phase, amplitude and polarization of the light beam for high quality focusing. In contrast with traditional objectives, they can be integrated into the sample chambers, thus allowing a more compact configuration and less drifting for OTs.

Several flat-lenses-based OTs have been proposed. For example, Shen et al. [21] have proposed a metalens enabling optical levitation in vacuum. Though it successfully levitates a nanoparticle with numerical aperture (NA) reaching 0.88 in air, its conventional designing strategy on the metalens leads to an equivalently reduced trapping stiffness and efficiency in aqueous medium, making it challenging to extend its applicability to the realm of biophysics research. Schonbrun et al. [22] and Markovich et al. [23] have demonstrated the optical trapping of polystyrene microspheres through the utilization of flat lenses with impressive NAs of 1.31 and 1.32, respectively. However, their achieved trapping stiffness remains comparatively modest and is difficult to be optimized since these lenses experience a notable reduction in deflection efficiency for larger deflection angles, thereby leading to a significant decrease in overall focusing efficiency [24]. Conversely, flat lenses characterized by lower NAs exhibit higher focusing efficiency while they are unable to attain the requisite trapping stiffness necessary for effective manipulation of biological objects [25], [26]. Therefore, flat lenses simultaneously with high NA and high focusing efficiency are indispensable for high-performance optical trapping. Very recently, Xiao et al. have demonstrated a metalens simultaneously achieving a high NA of 1.2 and high trapping stiffness up to 430 pN/(μm W) [27]. However, their metalens OT relies on a reflective configuration and circularly polarized light, which could potentially impose constraints on its broader applicability. For instance, numerous OT applications require the detection of transmitted light for force or torque measurement [28]–[30], as well as the utilization of light polarization for versatile optical trapping and manipulation [31]–[33]. For these experiments, a transmissive and polarization-insensitive metalens is much preferable. In addition to optical trapping, the capability of object manipulation is another essential function of OTs for various applications [34]. Interestingly, this aspect has rarely been addressed or demonstrated in prior studies employing metalens-based OTs. Consequently, the applicability of these metalens-based OTs still remains unclear.

**Figure 1:**
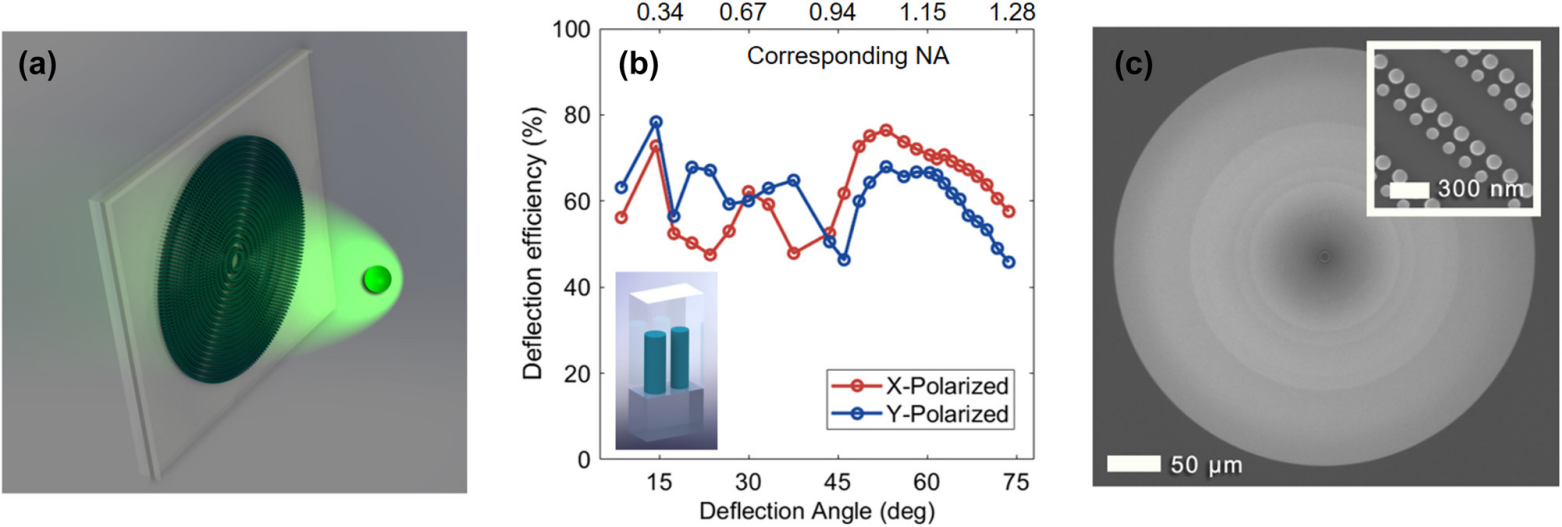
Characteristics of the metalenses and nano-antennas. (a) Schematic of the optical trapping by a metalens. (b) Deflection efficiency of the nano-antenna with different deflecting angles by *x*-polarized and *y*-polarized light beam. The inset is the sketch of the nano-antennas. (c) SEM images of the metalens. The inset shows the nano-antennas on the periphery of the metalens.

For these purposes, in this paper, a water-immersion metalens based on adaptive nano-antennas is designed for optical trapping and manipulating. The nano-antennas are formed as metagratings, which can achieve high deflection efficiency at large deflection angles by generating a continuous full 2π phase gradient for arbitrary linearly polarized incident beam even in aqueous environment. As a result, the metalens is of high NA (1.28) and is insensitive to polarization. We then experimentally demonstrate the high focusing efficiency (∼50 %) and stable optical trapping of polystyrene beads with ∼1.76 µm in diameter by using this water-immersion metalens [Figure 1(a)] at the wavelength of 532 nm. The measured lateral optical trapping stiffness is 534 ± 50 pN/(μm W) in *x*-direction and 668 ± 59 pN/(μm·W) in *y*-direction. To the best of our knowledge, such trapping stiffness is much larger than most reported results achieved by flat lenses, and it is comparable to the conventional OTs based on objectives with similar NAs. By using this metalens-based OTs, the bead can be laterally manipulated for more than 2 μm and the trapping stiffness remains approximately constant within a range of ∼0.5 μm. All these results provide an important guidance and foundation for applying metalens-based OTs to various prevalent OT experiments including single-molecule manipulation in the future.

## 2 Design and characterization of the metalens

Since many applications of OTs are in an aqueous environment, our metalens is designed to work under water immersion (refractive index 1.33). To achieve high focusing efficiency, the proposed metalens with the hyperbolic phase profile consists of polarization-insensitive nano-antennas [35] [Figure 1(b)], each of which is composed of crystalline silicon (c-Si) dimers with fixed diameters of 110 nm and 88 nm, respectively, and embedded in a protective silica layer (see Section 1 of Supplementary Material for the period and gap details of the nano-antennas). As shown in Figure 1(b), at the maximum deflection angle of 74° where the corresponding NA is 1.28 [36], the deflection efficiencies of the nano-antennas [37], [38] for *x*-polarized and *y*-polarized light beams are 58 % and 46 %, respectively. By using the method in a previous work [35], the simulated focusing efficiency of the metalens (55 µm in diameter) for the unpolarized incident beam is about 60 %.

The metalens with a diameter of 400 µm was firstly fabricated on a 270 nm-thick c-Si film of the silicon-on-insulator (SOI) wafer by electron beam lithography and then it can be obtained by transferring to a transparent silica substrate [39], as shown in Figure 1(c).

**Figure 2:**
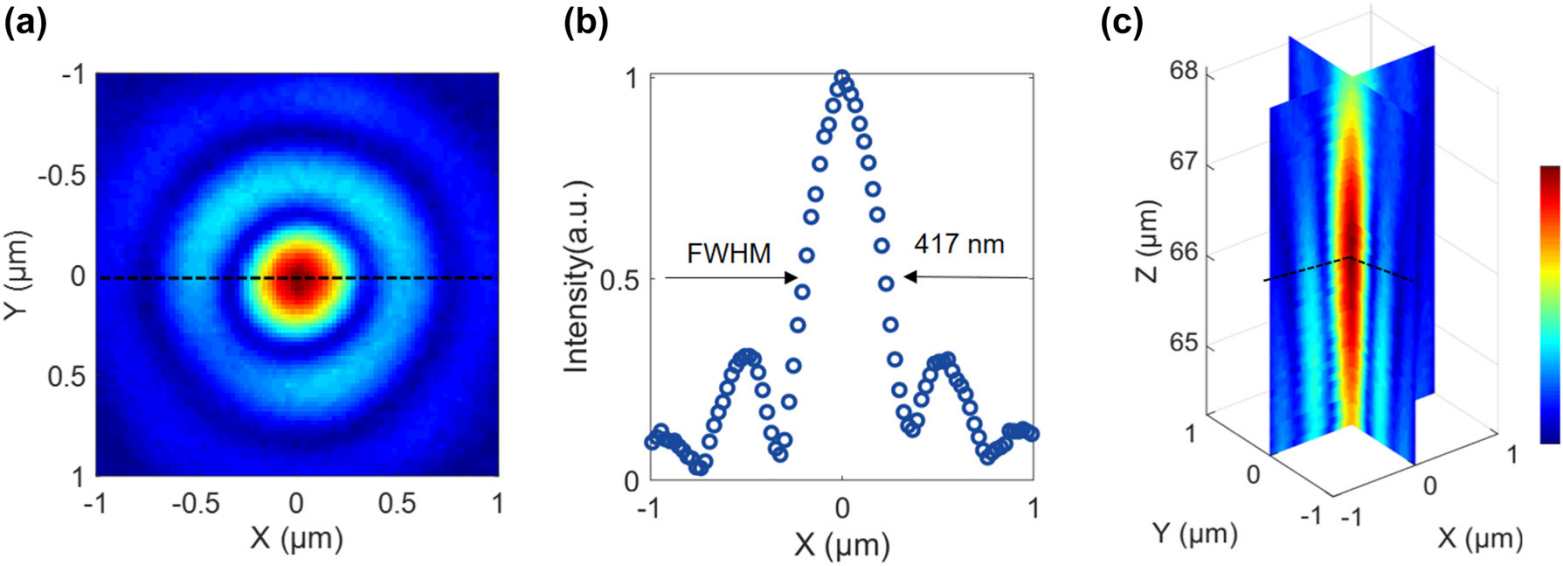
Experimental focusing performance of the metalens. (a) Experimental two-dimensional point spread function (PSF) of the metalens by an unpolarized incident light at the wavelength of 532 nm. (b) One-dimensional PSF along the black dotted line of (a). (c) The three-dimensional PSF to describe the experimental focal spot.

The focusing efficiency and the full width at half maximum (FWHM) of the focal spot (Figure 2) for the unpolarized incident beam were experimentally characterized (see Section 2 in Supplementary Material for the details of the optical setup). The focusing efficiency of the metalens reaches 50 %, through a circular aperture in the plane of focus with a diameter of 3 × FWHM [14], [17]. The FWHM was measured to be 417 nm, which was obtained from an Airy fitting to the one-dimensional distribution along the corresponding direction. Due to the restricted NA (1.2) of the objective in the current optical setup, the accurate measurement of the FWHM for the focus point is challenging. The actual size of the focus spot can be determined using the knife-edge method [40] or inferred from imaging with a confocal microscope system [35]. This result is larger than the theoretical value with the same NA and such a discrepancy mainly attributes to the phase mismatching between the ideal phase of the lens and the phase of the nano-antennas.

## 3 Optical trapping and manipulating performance

**Figure 3:**
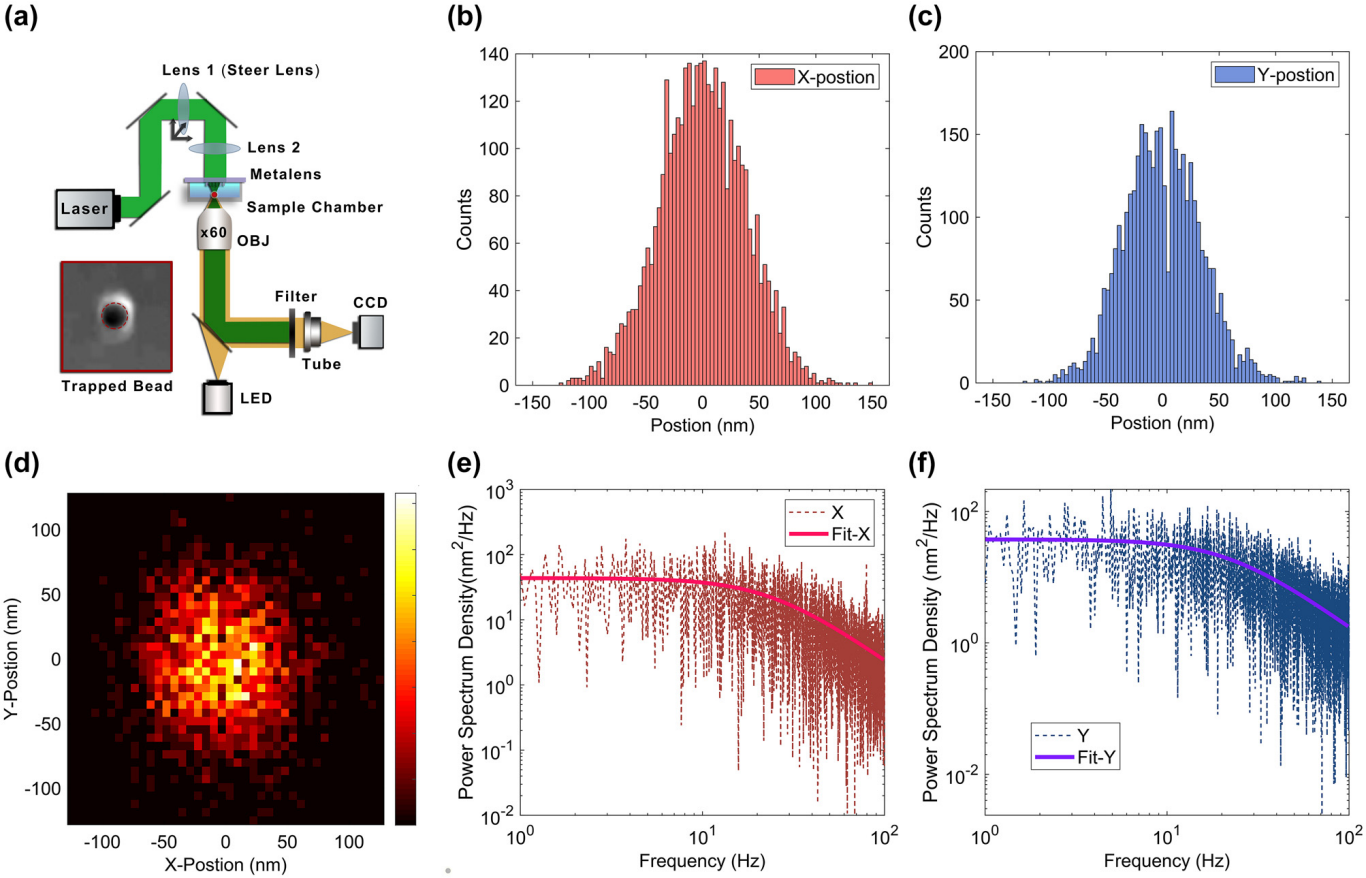
Experimental optical trapping performance of the metalens. (a) Schematics of optical trapping setup. The inset figure is the image of trapped bead in the sample chamber below the metalens. The position distribution of the trapped bead for the (b) X and (c) *Y* direction. (d) Spatial distribution for the trapping bead centers. The corresponding power-spectral-density (PSD) of the trapped bead for the (e) X and (f) *Y* direction fitted by a Lorentzian function.

To demonstrate the optical trapping and manipulating capabilities, a modified OT setup based on the metalens mentioned above was employed, as shown in Figure 3(a). In the optical trapping experiments, the polystyrene beads with an average diameter of 1.76 μm were utilized. This bead size is within the range of sizes of the commonly used beads in the single-molecule OT experiments, making the bead easy to be detected in our optical system. The input laser power was firstly set to be ∼30 mW for easiness to trap a bead in the flow, and then reduced to 5 mW in the following stiffness calibration. In this way, the corner frequency of the calibration signal was guaranteed to be less than half of the sampling frequency of the camera [41]. After that, a frame-by-frame analysis of the recorded video of the trapping bead at restricted Brownian motion state was performed. The recording time was set to be 10 s at a frame rate of 200 Hz. The image of each frame was mean-filtered and cross-correlated [42] to track the central position of the bead in motion. To quantitatively assess the trapping performance of the metalens, we measured the trapping stiffness by analyzing the Brownian motions of the trapped beads. Both the mean-square-displacement (MSD) analysis based on equipartition theorem [Figure 3(b–d)] and power spectral density (PSD) evaluation [Figure 3(e) and (f)] were employed for side-by-side comparison of the measured stiffness [6]. Detailed description for MSD and PSD analysis can be found in the Section 3 of Supplementary Material.

In our experiments, the measured trapping stiffness from MSD analysis are 534 ± 50 pN/(μm·W) and 668 ± 59 pN/(μm·W) at *x* and *y*-direction, respectively, while the corresponding values from PSD analysis are 501 ± 32 pN/(μm·W) and 628 ± 42 pN/(μm·W), respectively (see Section 4 of Supplementary Material for details). Both cases show good accordance with each other within the range of experimental errors. Table 1 shows the comparison of trapping performance from several reported works achieved by flat lenses. The present table is dedicated solely to the literature that reports on metasurface lenses applied in OT contexts; it does not encompass works involving metalens or planar lenses used for imaging [14], [16], [17], [19], [35], [41] or alternative application [43], [44]. As can be seen, the trapping stiffness obtained here is over one order of magnitudes larger than most of the previous reported results with flat lenses and also larger than the most recent result from Xiao et al. [27], thanks to both the high NA and high focusing efficiency of our metalens. The measured stiffness is also comparable to that from a conventional objective of similar NA, which is an essential step for applying the metalens-based OT to the prevalent OT applications including single-molecule studies. It is worth noting that the averaged diameter of the beads used in our optical trapping experiment is 1.76 μm, which is smaller than those used in other studies listed in Table 1. However, when performing numerical simulation to evaluate the trapping stiffness variation with different sizes of the beads (Section 5 of Supplementary Material), we found that the optical trapping stiffness for beads with the diameters of 1.76 μm is only ∼13 % larger than that for 2 μm, and about 156 % larger than that for 4.5 μm. It’s worth noting that this simulation result is consistent with previous reports. The fact that trapping stiffness decreases monotonously with the particle size within the range of 1–5 μm in diameters can be attributed to the averaging of the intensity gradients over sphere volumes larger than the dimensions of the focus, and the compensation of the increasing repelling force from the lateral scattering [45]. Therefore, the substantial increase in trapping stiffness observed in this study cannot be solely attributed to the variation in bead sizes employed during the experiments.

**Table 1:** Summary of previously reported experimental trapping performance of flat lenses in aqueous medium.

Reference	NA	Polarization	Wavelength (nm)	Beads type	*Kx* pN/(μm·W)	*Ky* pN/(μm·W)	Calculation method
This work	1.28	Polarization insensitive	532	1.76 µm diameter polystyrene	534	668	MSD
				beads	501	628	PSD
Schonbrun et al. [22]	1.31	Linear polarization	976	2 µm diameter polystyrene beads	29.4	27.7	MSD
Markovich et al. [23]	1.32	Linear polarization	980	2 µm diameter polystyrene beads	6.5	6.9	MSD
Tkachenko et al. [24]	0.74	Linear polarization	1064	2 µm diameter polystyrene beads	13.5^a^	33.7^a^	MSD
Plidschun et al. [25]	0.88	Elliptically polarization	660	2 µm diameter silica beads	103.8^b^		PSD
Chantakit et al. [26]	0.60^c^	Circular polarization	800	4.5 µm diameter polystyrene beads	9.5^b^		PSD
Xiao et al. [27]	1.20	Circular polarization	830	2 µm diameter latex beads	430^a,b^		PSD
Conventional objective	1.20	Polarization insensitive	532	1.76 µm diameter polystyrene	1097	1291	MSD
				beads	888	1108	PSD

^a^The flat lens OT is based on a reflective configuration. ^b^The reference reported the transverse trapping stiffness. ^c^The reference reported the NA in air and the polystyrene beads in aqueous environment.

**Figure 4:**
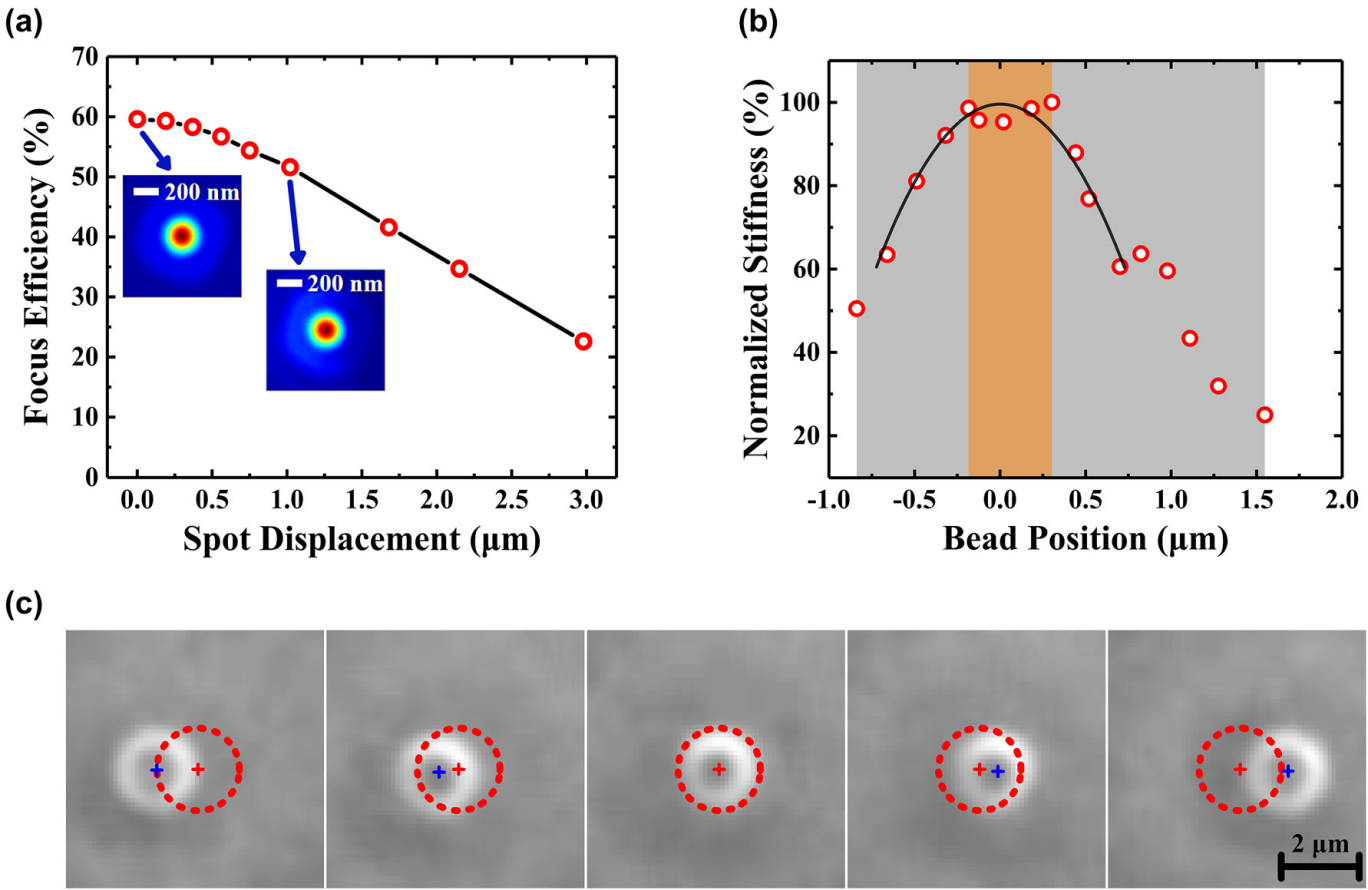
Experimental optical manipulating performance of the metalens. (a) The simulated focusing efficiencies for different trap displacements. The inset images are PSF at normal incidence to the metalens and PSF at 1 µm displacement. (b) Stiffness as a function of the bead position during optical trap steering. The solid curve is a second order polynomial fitting for the stiffness points and the center of the fitting curve is set as the zero point of the bead position. The steering region with the stiffness variation <5 % and the total steering region are highlighted in orange and gray, respectively. (c) The image of trapped bead steered with the optical trap on *X* axis. Red dotted circles indicate the same location for the bead with maximum trapping stiffness. Red cross and blue cross indicate the center of the red circle and the center of the tracked bead, respectively.

Furthermore, many applications of OTs involve not only effective trapping but also the manipulation of small objects [46]–[48]. To address this, we extended our investigation to demonstrate the manipulation capabilities of our metalens-based OTs through a bead-steering experiment. As depicted in Figure 3(a), altering the position of Lens 1 along the optical path modifies the incident angle of the beam onto the metalens, thereby causing the focal spot (i.e. trap) to shift in a translational manner. In order to increase the translational displacement, the tiled angle of the input laser beam needs to be increased. At this point, due to the strong off-axis aberration of our single layer metalens with hyperbolic phase profile for the obliquely incident light, it leads to a deterioration in the focal spot and a decrease in focusing [49], which inevitably reduce the trapping stiffness with this increasing translational displacement. To assess the range of effective trapping, we initially conducted numerical simulations. Figure 4(a) shows the calculated focusing efficiency in relation to focus displacement. Additionally, the simulated PSFs for scenarios involving normal incidence and a 1 µm displacement are also presented in Figure 4(a). The outcomes reveal that the maximum focusing efficiency reaches 60 %, occurring at normal beam incidence onto the metalens. This efficiency progressively decreases with an increase in translational displacement.

In the bead-steering experiment, the bead was effectively trapped and manipulated by adjusting the translational stage for Lens 1 while utilizing a laser power of 5 mW. Interestingly, elevating the laser power to 18 mW did not noticeably extend the range of mobility within our experimental setup. Subsequently, the stiffness of the bead’s trapping at different positions was determined via the MSD method, with the results graphically displayed in Figure 4(b). The effectiveness of trapping and manipulating of beads are clearly shown in Figure 4(c), as well as Visualization 1 and Visualization 2.

As observed, the stiffness of the bead exhibits minimal variation (<5 %) within the approximate range of ∼±0.25 μm surrounding the central position [marked as the orange region in Figure 4(b)]. In contexts where precise quantitative experiments are required, such as employing dual optical trap assays for the stretching of a single DNA molecule to measure its force-extension curve [50], maintaining a relatively stable trap stiffness during trap steering is crucial. This scenario could be effectively realized utilizing our metalens-based OTs, provided that the extent of trap movement remains confined within the delineated orange region in Figure 4(b).

However, when venturing beyond the orange region in Figure 4(b), the stiffness of the optical trap diminishes with the increment of bead displacement, as indicated within the gray region in Figure 4(b). For instance, the measured trapping stiffness experiences a decline to approximately 40 % of its maximum value when the trap’s position shifts from the central point to a 1 μm displacement [as shown in Figure 4(b)]. As confirmed by Figure 4(a), we posit that this reduction in optical trap stiffness results from the diminishing focusing efficiency [51], [52], rather than being attributed to any coma aberration stemming from the metalens. Beyond the gray region in Figure 4(b), the bead would eventually escape the optical trap. Remarkably, the maximal maneuvering range for a bead was ascertained to exceed 2 μm.

It is essential to note that the demonstrated steering range is already applicable to a broad spectrum of single-molecule experiments [29], [53]–[55]. Furthermore, this steering range can be augmented by refining the capabilities of the metalens. For example, by employing a metalens array [43], [44] to generate multiple focus spots, the movement of the trapped object can be suitably relayed to effectively expand its range of motion. A similar approach has already been successfully showcased in the realm of nano-photonic waveguides, specifically through the nano-photonic standing-wave array trap system [9], [10]. Furthermore, although the axial manipulating has not been demonstrated in our bead-steering experiment, *z*-direction control can be potentially achieved by slightly adjusting the steering lens to tune the convergence of the laser beam [6] or utilizing the chromatism of the metalens [56] and tune the wavelength of the laser to get different focal lengths (see Section 6 of Supplementary Material). However, our metalens-based OT is expected to employ dual optical trapping assays. Such an assay only requires the lateral manipulating and force measurement and has been widely used in single-molecule experiments to study various biological processes, such as DNA mechanics, RNA or protein folding, protein-nucleic acid interactions, etc. [57–60]. For this purpose, the ability of lateral trapping and manipulating has been our focus in the bead-steering experiment.

## 4 Conclusions

We have demonstrated a transmissive-type and polarization-insensitive water immersion metalens simultaneously with high NA and high focusing efficiency for optical trapping and manipulation. The achieved trapping stiffness is on the same order of magnitude as the conventional objective-based OTs and much larger than most of previously reported values for metalens-based OTs. This metalens is beneficial to the advance of integration and performance of flat-optics-based OTs from the following aspects: firstly, it can be easily integrated into the sample chamber so that the size of the OT can shrink into a compact flat-optics-based volume. Secondly, its integrated configuration is inherently stable to keep the trapping position from drifting away from the chamber surface, getting rid of the complicated drift-reduction complements. Thirdly, our metalens is transmissive type, which is the same as an objective, making it compatible with most conventional OT applications. For example, in a typical single-molecule experiment with an OT, the transmitted light needs to be detected for force [61], [62] or torque [30], [63] measurement. OTs based on a reflective-type metalens would be difficult to separate the transmitted light from the incident light. Fourthly, the trapping performance of our metalens approaches the level of conventional objectives, making it ready for many prevalent OT applications including single-molecule or single-cell experiments. Finally, the polarization insensitive nature of our metalens is especially useful when the polarization of the light is utilized for some special OTs, such as angular optical trap [28], [64] and other torque wrenches [65], [66], as well as various OTs involving using vector beams [67]–[69]. This is in sharp contrast with most of the reported metalens which can only response to a particular polarization state (see Table 1).

Additionally, we show that our metalens-based OT can achieve more than 2 µm manipulation distance and ∼0.5 µm moving range with approximately constant stiffness. Though these values are relatively small in comparison with conventional objective-based OTs, they are still acceptable for many experiments, e.g., single-molecule experiments with short (several hundred- to kilo-base pairs) DNA samples. This also points out one of the directions to further improve our metalens in the future.

As a summary, our metalens is transmissive and polarization-independent, able to generate large optical force due to its high stiffness and potentially allows diversified operations in manipulation, enabling a novel, flexible and reliable high-performance flat-optics-based OT for a wide variety of studies.

## Supplementary Material

Supplementary Material Details
