# Supplementary material for: Optical trapping and manipulating with a transmissive and polarization-insensitive metalens
Source: Nanophotonics. 2024 Apr 15;13(15):2781–9. doi: 10.1515/nanoph-2023-0850 (PMC11501135; doi:10.1515/nanoph-2023-0850)
Supplement: Supplementary file 1 — Supplementary Material Details [file j_nanoph-2023-0850_suppl_001.pdf]

Supplementary Material:

Optical trapping and manipulating with a transmissive and polarization-insensitive metalens

Dongni Yang,[#] Jianchao Zhang,[#] Pengshuai Zhang, Haowen Liang,[*] Jie Ma,[*] Juntao Li,[*] and Xuehua Wang

AUTHOR ADDRESS

State Key Laboratory of Optoelectronic Materials and Technologies, School of Physics, Sun Yat-Sen University, Guangzhou 510275, China

1. Nano-antennas Cell Parameters

The nano-antenna is comprised of two pillars in a periodic unit, as shown in Fig. S1. The period of these units in the radial direction (P_{radial}) is determined by the grating equation:

$$P_{radial}(r) = \lambda/(n_g \sin\theta\,(r)) \quad \text{(S1)}$$

where $\theta(r)$ is the target deflection angle, which is a function of metalens' radius r. According to the Figure S1, the parameters to optimize are the height, the gap between the pillars, the period in azimuthal direction ($P_{angular}$) and the diameters of the two pillars (D_1, D_2).

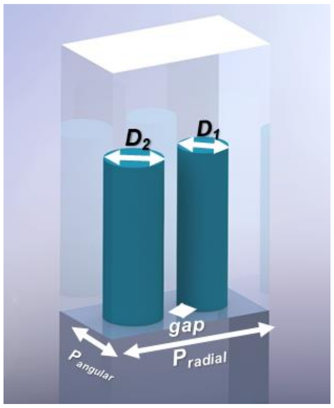

Fig. S1. Sketch of the nano-antenna, which consists of two circular pillars with a height of 270 nm.

Table S1. Nano-antennas Parameters

Deflection Angle (deg)	Gap [a] (nm)	Pangular [b](nm)	Pradial [c](nm)
73.74	50	220	417
71.81	50	220	421
68.44	50	220	430
66.93	50	220	435
64.16	50	200	444
61.64	50	195	455
58.21	50	195	471
53.13	50	185	500
48.59	50	170	533
44.43	50	170	571
33.37	85	170	727

26.74	120	170	889
20.49	175	170	1143
14.48	250	170	1600
8.63	400	170	2667

[a] The distance between the two cylinders that make up the nano-antennas.

[b] Nano-antennas' period along the angular direction of the metalens.

[c] Nano-antennas' period along the radial direction of the metalens.

2. Experimental setup for the focal spot measurement

As shown in Fig. S2, the experimental focusing performance is characterized by a microscopic imaging system, including a 60× water immersion objective lens (Nikon CFI Plan Apochromat VC 60XC WI), a tube lens (LBTEK MCX10619-A), a laser source at wavelength of 532 nm and a CCD camera (Kiralux CS895CU). We generate unpolarized beam with a depolarizer (LBTEK APD10, DP). The tube lens has a focal length of 50 cm, which leads to a 150× system magnification. The actual size of focal beam intensity distribution can be calculated by dividing the recorded spot size from the CCD camera by this system magnification.

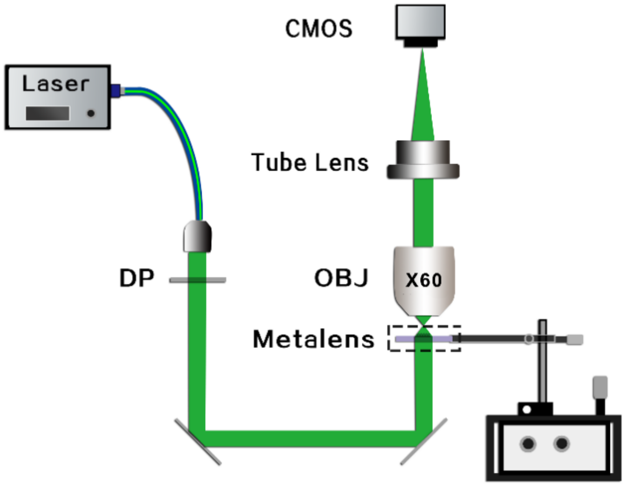

Fig. S2. Schematic diagram of the metalens focal spot measurement for unpolarized incident laser beam.

3. Mean-Square-Displacement (MSD) and Power Spectral Density (PSD) Methods for Stiffness Measurement

For the MSD methodl, the distribution probability of the trapped bead is statistically analyzed, and then the variance $\langle x^2 \rangle$ of the x-displacement of the bead from its equilibrium position can be obtained, which follows the equipartition theorem:

$$\frac{1}{2} k_B T = \frac{1}{2} \kappa_{MSD} \langle x^2 \rangle$$

where k_B is the Boltzmann's constant, T is the absolute temperature and κ_{MSD} is the trapping stiffness calculated from MSD analysis.

For the PSD methodl, the one-sided power density spectrum $S_x(f)$ of the x-displacement of the bead can be described by the Lorentzian profile as:

$$S_x(f) = \frac{k_B T}{\pi^2 \beta (f^2 + f_c^2)} \qquad \text{(S3)}$$

where β is the hydrodynamic drag coefficient of the bead and f_c is the corner frequency, which are associated with the trapping stiffness by

$$\kappa_{PSD} = 2\pi\beta f_c \qquad \text{(S4)}$$

From Equation (S3), we can define the parameter A as:

$$A = \frac{k_B T}{\pi^2 \beta} \qquad \text{(S5)}$$

In this case, the parameter A and the corner frequency f_c can both be obtained from fitting the power density spectrum $S_x(f)$. Thus, the trapping stiffness can finally be determined from Equation (S3) – (S5) as:

$$\kappa_{PSD} = \frac{2 k_B T}{\pi A} f_c \qquad \text{(S6)}$$

4. Results of the Measured Stiffness

The measured trapping stiffness results for 36 individual beads with the averaged diameter of 1.76 μm at X and Y-direction using MSD and PSD method are shown in Table S2 as follow.

Table S2. Results of the measured stiffness for 36 individual beads.

MSD [pN/(μm·W)]		PSD [pN/(μm·W)]	
X	Y	X	Y
566	689	494	626
590	706	510	680
548	686	506	693
452	682	440	587
562	775	475	634
526	679	524	645
617	686	544	596
641	715	573	625
433	688	492	651
512	737	525	614
470	625	522	697
553	760	485	640
492	653	465	681
479	693	480	715
414	644	518	565
534	639	446	683
492	739	471	629
549	595	452	548
521	591	472	603
556	654	481	547
542	671	483	632
547	734	531	629
495	696	496	634
544	747	511	598
604	639	538	610
554	591	527	543
509	508	446	580
507	619	499	627
506	594	500	614
555	569	477	616
543	591	493	608
473	731	555	682

561	689	505	637
601	648	548	663
580	702	529	651
581	688	522	624

5. Simulation of the Optical Trapping Stiffness

The theoretical lateral stiffness in an optical trap varied with different bead sizes is simulated using an open-source software optical tweezers computation toolbox in Matlab published previously2. A linear polarized Gaussian beam source with wavelength of 532 nm and numerical aperture (NA) of 1.28 is used to match the parameters in our optical trapping experiments. The shape of the trapped bead is chosen as the ideal sphere, with the refractive index of the polystyrene with the value of 1.59. Bead sizes are varied from 1 μm to 4.5 μm in diameter. The refractive index of the medium is 1.33. Lateral force curves versus lateral bead displacements can be calculated for beads of different sizes and normalized by the power of the beam source, and the linear fitting of the slope for the proportional region of the lateral force curves yields the theoretical lateral stiffness, as shown in Fig. S3.

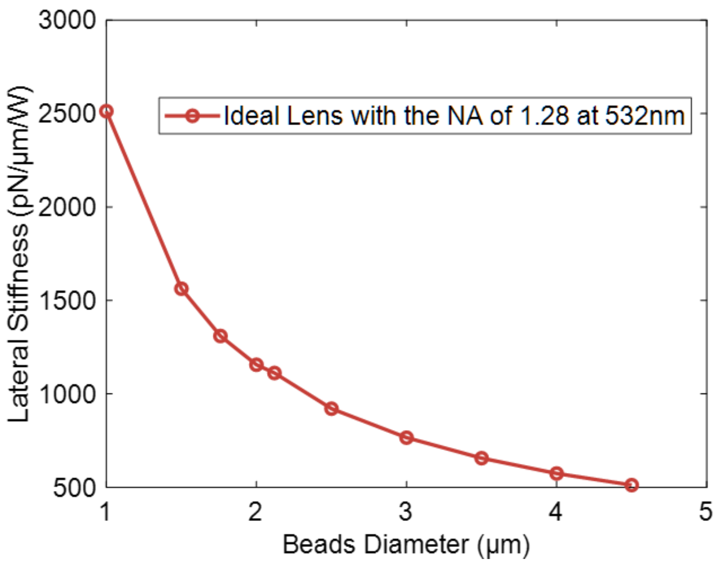

Fig. S3. The simulated optical trapping stiffness of the ideal objective or metalens with the NA of 1.28 for beads of different diameters at 532 nm.

6. Simulation of Metalens under Various Incident Wavelengths

We could utilize the chromatism of the metalens and tune the wavelength of the laser to get different focal lengths and realize z-direction control of the trap. Therefore, we simulate the focal lengths of the metalens under various incident wavelengths. The simulated metalens has a diameter of 55 μm. When the incident light wavelength ranged from 527 nm to 545 nm, all the focusing efficiencies of the metalens exceed 50%, which is conducive to robust trapping of particles. Within this spectral range, the corresponding focal length varied between 6.9 μm and 7.9 μm, as shown in Fig. S4. When the diameter of the simulated metalens is scaled up to match that of the experimental sample (400 μm in diameter), it is anticipated that the focal length could vary within a range of 50 μm to 57 μm. Consequently, it is predicted that three-dimensional optical manipulation could be realized within a z-axis range of 50 ~ 57 μm.

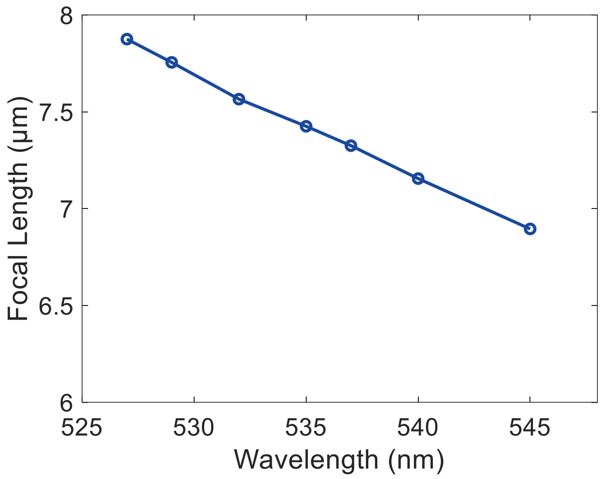

Fig. S4. The relationship between the metalens focal length and the laser beam wavelength.

References for Supplemental Information

[1] K. C. Neuman and S. M. Block, "Optical trapping," Rev. Sci. Instrum., vol. 75, pp. 2787-2809, 2004.

[2] T. A. Nieminen, V. L. Y. Loke, A. B. Stilgoe, G. Knoner, A. M. Branczyk, N. R. Heckenberg, and H. Rubinsztein-Dunlop, "Optical tweezers computational toolbox," J. Opt. A-Pure Appl. Op., vol. 9, pp. 196-203, 2007.
